# Current status and future perspectives for sequencing livestock genomes

**DOI:** 10.1186/2049-1891-3-8

**Published:** 2012-03-01

**Authors:** Yongsheng Bai, Maureen Sartor, James Cavalcoli

**Affiliations:** 1 Center for Computational Medicine and Bioinformatics and Department of Computational Medicine and Bioinformatics, The University of Michigan, 100 Washtenaw Ave., 2017 Palmer Commons, Ann Arbor, Michigan 48109-2218, USA; 2 Department of Biostatistics, The University of Michigan, 1415 Washington Heights, Ann Arbor, MI 48109-2029, USA

**Keywords:** livestock genomes, next-generation sequencing technology, nutrition

## Abstract

Only in recent years, the draft sequences for several agricultural animals have been assembled. Assembling an individual animal's entire genome sequence or specific region(s) of interest is increasingly important for agricultural researchers to perform genetic comparisons between animals with different performance. We review the current status for several sequenced agricultural species and suggest that next generation sequencing (NGS) technology with decreased sequencing cost and increased speed of sequencing can benefit agricultural researchers. By taking advantage of advanced NGS technologies, genes and chromosomal regions that are more labile to the influence of environmental factors could be pinpointed. A more long term goal would be addressing the question of how animals respond at the molecular and cellular levels to different environmental models (e.g. nutrition). Upon revealing important genes and gene-environment interactions, the rate of genetic improvement can also be accelerated. It is clear that NGS technologies will be able to assist animal scientists to efficiently raise animals and to better prevent infectious diseases so that overall costs of animal production can be decreased.

## 1. Current Status of Domestic Animal Reference Sequences

As the new genomics era matures, with large-scale genome research and the development of sophisticated bioinformatics tools that can be applied to the agricultural field, agricultural researchers should take advantage of and benefit from new sequencing and mapping technologies. In recent years, the genomes of several domesticated livestock animals (chicken, pig, cow, sheep, and horse) have been partially or completely sequenced. In this review, we first examine the current sequencing status for several sequenced agricultural species. Next, we discuss the different platforms used for genome sequencing, tools available for mapping sequences to the genome, and several additional applications for which next generation sequencing can be used. We also list tools available for analyzing data from these additional applications.

Due to the high recombination rate of its micro-chromosomes, the chicken is an ideal model for studying genetic linkage [1]. The chicken genome sequence of Red Junglefowl (RJF) was the first livestock species to be sequenced. The first draft of the chicken genome was built from an assembly with 6.6-fold whole-genome shotgun coverage, although sex chromosomes were poorly annotated in the initial assembly [1,2]. The updated version of NCBI build 2.1 was released recently with a significant improvement on the annotation of the sex chromosomes. Roughly 2.8 million SNPs for chicken were identified [1,3,4] between the base (wild type) RJF sequence assembly and a partial genome scan of three chicken breeds: a female layer (White Leghorn); a male broiler (Cornish); and a female Silkie. A moderate density (60 k) Illumina SNP BeadChip for commercial chicken (broilers and layers) containing 352,303 SNPs was designed and additional SNPs not covered by the current chicken genome assembly (Gallus_gallus-2.1) were identified and selected recently [5]. The BBSRC ChickenEST Database (http://www.chick.manchester.ac.uk/) provides the most comprehensive database [6,7] of ESTs/cDNAs for the chicken genome. Chicken Variation Database (ChickVD) (http://chicken.genomics.org.cn/) was released in 2005 [4] for geneticists to use, and contains the genes, variants, chicken orthologs of human disease genes, and QTLs which are stretches of DNA containing or linked to the genes that underlie a quantitative trait. Large scale breeding research projects are still needed (http://www.nih.gov/science/models/gallus/).

In November 2009 the first draft (98% complete) of the pig genome (*Sus scrofa*) assembled from global collaborative efforts was released. The diploid pig genome has 38 chromosomes (including meta- and acrocentric ones) and is roughly 2.7 × 10^9 ^bp. Both high-throughput fingerprinting and BAC (bacterial artificial chromosome) end sequencing over 600,000 BAC end sequences) were used as templates for sequencing the whole swine genome. Specifically, the restriction enzyme fingerprinting method [8] was used to construct a physical map through bacteria-based clones for the swine genome. The sequence will be used as the basis to identify genes that are important to pork production and/or are involved in immune or physiological processes (http://www.sanger.ac.uk/about/press/2009/091102.html). The finished pig assembly will not only help researchers to understand its genetic complexity, but it will also change pork production and breeding technology. The completed swine genome is critical to helping researchers study human nutrition and disease, due to these animals' similar physiology and nutritional needs to humans (http://www.sanger.ac.uk/).

The genome sequence of Taurine cattle was initially sequenced and assembled with approximately 7-fold coverage and was published by the Bovine Genome Sequencing and Analysis Consortium in April 2009. This initial assembly reported roughly 22,000 genes and 14,345 orthologs shared among seven mammalian species [9]. Bovine Genome Sequencing Projects led by the Baylor College of Medicine Human Genome Sequencing Center in Houston, Texas released an improved assembly version (Btau_4.2) for the cow genome in 2009. The BCM4 assembly was constructed using the Atlas assembly program [10]. The assembly of UMD2 from Steven Salzberg and his colleagues in Baltimore, Maryland was constructed using NCBI traces and strengthened using several modified, powerful assembly and mapping tools. Roughly 24 million reads from whole genome sequencing and 11 million reads from BACs were used to create the UMD2 assembly [11]. The Salzberg lab recently created an updated assembly (UMD3.1) of 2.86 billion base pairs with 9.5x coverage of the genome [11]. Even with all of these efforts that researchers have invested, the cow genome is still not completely assembled. The Illumina BovineSNP50 is a high-density, genome-wide genotyping array. The v2 Bead Chip contains 54,609 SNPs of major breed types. The probes were validated in 19 common beef and dairy breeds. This makes certain types of research, such as QTL discovery and genetic improvement possible (http://www.illumina.com/products/bovine_snp50_whole-genome_genotyping_kits.ilmn). Although BovineSNP50 was successfully used, several new chips have been designed and/or are being designed. Besides keeping BovineSNP50 SNPs, Bovine High-Density (HD) Bead Chip (778K SNP) includes some Y-specific and mitochondrial SNPs. Other chips, such as Bovine Low-Density (3K) Bead Chip, 96 SNP parentage chip, 384 SNP chip, and 700 K SNP Affymetrix chip were designed to use for different genetic purposes (http://www.slideserve.com/Download/143258/Walking-the-Cattle-Continuum-Moving-From-the-BovineSNP50-to-Higher-and-Lower-Density-SNP-Panels). A new collaborative project between Australian beef and dairy industries and international partners is constructing a database of functional polymorphisms and sequence information on 1,000 cattle. This will facilitate research on identifying features in the genome that are related to economically important traits (http://www.beefcrc.com.au/Assets/819/1/BeefBulletin-September20117-9-11webspreads.pdf). Given the importance of the Bovine sequence in impacting the dairy industry's genetic gains, future technology and novel assembly methods are desired to bring the cow genome annotation to a more complete state and to provide a faster, cost-efficient way of sequencing other cattle breeds. Such sequencing projects could help understand variation in resistance to disease and lead to improved breeding programs.

The interim assembly version OARv2.0 for sheep was released recently [12] with the goal of identifying genes associated with production, quality, and disease traits in sheep (http://www.sheephapmap.org/). The OARv3.0 is projected to be released in late 2011 with the expected improvement that chromosomal gaps will be filled and many of the unassigned sequences in v2.0 will be correctly assigned to chromosomes. In addition, transcriptomic and SNP datasets are expected in the new release as well (http://sheephapmap.org/news/Scheduled_OARv3.php).

The horse is a model organism for studying biomechanics and exercise physiology (http://www.ncbi.nlm.nih.gov/projects/genome/guide/horse/). The sequence of the horse is also important to help veterinarians study new therapies for horse laminitis and respiratory diseases. In recent years, there has been progress in the identification of mutations in genes related to morphology, immunology, and metabolism in the horse [13].

The detailed sequencing description for the above mentioned domestic animals is listed in Table 1.

**Table 1:** Various sequenced livestock genomes

Animal	Species	Genome size	Sequencing methods	Recent release version	Sequencing center
Chicken	Gallus gallus	1.2 Gb (39 chromosome pairs)	Bacteria Artificial Chromosomes (BAC), fosmid, and plasmid-based whole genome shotgun (WGS)	NCBI build 2.1	Washington University Genome Sequencing Center
Pig	Sus scrofa	2.7 Gb (18 autosomes, X and Y sex chromosomes)	Clone based	NCBI build 3.1	The Swine Genome Sequencing Consortium
Cow	Bos taurus/B.indicus	2.86 billion base pair	Mixture of hierarchical and whole-genome shotgun	UMD_3.1	The original sequencing was conducted at the Baylor College of Medicine in Houston, Texas, but the genome was reassembled by Salzberg lab in Baltimore, Maryland
			7.15x mixed assembly of whole-genome shotgun and BAC sequence	Btau_4.2	Bovine Genome Sequencing Project led by the Baylor College of Medicine's Human Genome Sequencing Center in Houston, Texas
Sheep	Ovis aries	2.71 Gb (91% of sheep genome)	WGS	OARv2.0 (working draft)	International Sheep Genomics Consortium
Horse	Equus caballus	2.4-2.7 Gb	6.79x WGS	EquCab2.0	The Broad Institute and the Horse Genome Project

## 2. Next Generation Sequencing Technologies

Next generation sequencing technologies (NGS), using modern methods/platforms to produce significant numbers of sequence fragments, have revolutionized research in genetic and biomedical fields and have become increasingly popular in recent years. Several massively parallel platforms are in widespread use by sequencing centers or laboratories at present. These include the Illumina (former Solexa) Genome Analyzer, HiSeq (http://www.illumina.com), Roche/454 FLX (http://www.454.com), and the Applied Biosystems SOLiD™ System (http://www.appliedbiosystems.com). These platforms can generate millions to billions of reads in a single run with the read length in the range of 50 to 500 bp. The difference between these technologies is embodied in many parameters such as clonal amplification method, instrument used, sequencing enzyme/method used, and read length generated. Since the number of reads produced and sequencing speed differ among technologies, the generation rate is also different among these technologies. Current Illumina HiSeq technology can generate 150 to 200 Gb data for paired-end 100 bp read length in 8 days. The base call accuracy also varies between these platforms (http://kevin-gattaca.blogspot.com/2010/04/comparing-ngs-platforms-454-solexa.html).

Several cutting-edge biological applications such as targeted exome capture or exome sequencing, Chromatin Immunoprecipitation sequencing (ChIP-Seq), and whole transcriptome shotgun sequencing technology or RNA-Seq have been developed to fulfill different biological purposes. Exome-sequencing [14] overcomes the drawback of the high cost of sequencing the whole genome by excluding intronic regions and selectively sequencing the exonic regions that might be of more immediate interest. ChIP-Seq [15] is used to identify genome-wide binding patterns of a protein of interest such as a transcription factor and is a powerful approach to study protein-DNA/RNA interactions. RNA-Seq [16,17] or transcriptome-wide sequencing is used to exploit NGS technologies to sequence cDNAs from RNA samples.

To reveal variations among different strains or large populations of related samples, one of the above NGS techniques can be employed because of its advantages, such as a high efficiency to cost ratio (according to the National Human Genome Research Institute (NHGRI) (http://genome.gov/sequencingcosts)). The cost per megabase of DNA sequencing was under 50 cents and cost per genome was estimated at $11,000 in March 2011. Sequence mutation and structure variations are commonly searched in the targeted sequencing (exome or whole genes). Popular SNP detection tools are SNVMix [18], SAMtools [19], and GATK application package [20,21]. Structure variation (copy number variation) detection tools/methods, such as CNV-seq [22], SLOPE [23], SVDetect [24], and associated statistical methods have been developed in recent years to identify INDELs, tandem duplications, and other genetic variations.

RNA-Seq technology is being used as a popular method for quantitative gene expression studies [25]. However, accurate gene expression estimation requires accurate genome annotation [26]. By utilizing complete or nearly completely annotated reference genomes, RNA-Seq can assist researchers to identify differentially expressed genes and novel transcripts for agricultural animals in a quantitative and efficient way. The power of RNA-Seq is not only in helping agricultural researchers to select differentially expressed genes between samples under different treatment condition(s) that could be crucial for certain traits or disease resistance, but it can also reveal multiple isoforms that template assembly does not possess in its annotation. There are several popular differential expression testing tools for RNA-Seq data, such as edgeR [27] and DEGSeq [28]. Powerful splice junction sites identification tools are represented by Cufflinks [29]/TopHat [30] and Supersplat [31]. RNA-Seq technology can also assist researchers in annotating transcription of the genome in a complete manner at different developmental stages [26].

A collection of current popular NGS tools/algorithms and their description in fulfilling the goals for different biological applications is listed in Table 2.

**Table 2:** Selected variant calling, RNA-Seq, and ChIP-Seq software/tools and database links

Name	Description	Features/Restrictions	Link
SNVMix	Detects single nucleotide variants from next generation sequencing data	Input files are Maq or Samtools pileup format	http://www.bcgsc.ca/platform/bioinfo/software/SNVMix
SAMTools	Manipulating alignments in the SAM format (sorting, merging, indexing and ...)	The software is free and is designed for multiple uses.	http://samtools.sourceforge.net/
GATK	Contains modules of depth of coverage analyzers, quality score recalibrator, SNP/Indel caller, and local realigner	The software is Java based and requires input files as sorted, indexed BAM alignment files and a fasta-format reference with associated index files	http://www.broadinstitute.org/gsa/wiki/index.php/The_Genome_Analysis_Toolkit
ERANGE (RNA-Seq)	ERANGE is a python package and uses the Cistematic package	The software is free and gives the flexible input parameter choice	http://woldlab.caltech.edu/rnaseq/
Illumina (RNA-Seq)	Counts can be visualized and analyzed in Illumina's GenomeStudio viewer	License required, more robust (requires Illumina's output directory contents)	http://www.illumina.com/
TopHat	Fast splice junction mapper	Input files can be either FASTQ or FASTA format	http://tophat.cbcb.umd.edu/
Cufflinks	Assembling transcripts and estimating their abundances from RNA-Seq data	Input alignment files are in the SAM format and the software requires reference annotation GTF file	http://cufflinks.cbcb.umd.edu/
ERANGE (Chip-Seq)	Studying protein-DNA interactions	Free	http://woldlab.caltech.edu/erange/README.chip-seq
HPeak	The software can accurately pinpoint regions to which significantly more sequence reads are mapped	Hidden Markov model-based approach	http://www.sph.umich.edu/csg/qin/HPeak/
MACS	Uses a dynamic Poisson distribution to effectively capture local biases in the genome sequence and allows for more sensitive and robust prediction	The software is publicly available open-source, and used for ChIP-Seq analysis with or without control samples.	http://liulab.dfci.harvard.edu/MACS/
CISGenome	An integrated tool for tiling arrays, ChIP-seq, genome and cis-regulatory element analysis	N.A.	http://www.biostat.jhsph.edu/~hji/cisgenome/

## 3. Challenges and Perspectives for Livestock Sequencing Research

From raw draft assembly to full length cDNA/EST resources and BAC libraries, livestock species have undergone significant annotations in recent years. The consequence of sequencing agricultural animals has expanded far beyond the original goals of serving as a model for studying human health issues and physiological phenomena, to increasing our understanding of the human genome, and to studying traits of economic and biological interest to raising livestock production. We are now at the beginning of an era where genome sequencing analysis of livestock will allow study of domestication, selection of better breeds (e.g. high fertility) and understanding of quantitative differences due to environmental factors (e.g. nutrition). Gene-gene and gene-environment interactions related to environmental conditions could be studied quantitatively using modern bioinformatics tools. It can clearly be seen that sequencing individual animal genomes or interesting regions under different treatment conditions will benefit the agricultural community by providing guidance for experimental design and animal disease control and prevention. Livestock animals serve as a major meat/egg/dairy (protein) source for human beings. The need to reduce the use of chemicals/antibiotics and improve genetic resistance to pathogens is becoming increasingly important to human beings and agricultural scientists [1]. These new goals are too time consuming and/or costly to be achieved using traditional genetic approaches. NGS technologies will enable a breakthrough in genetics studies by shortening the sequencing time and decreasing the cost. NGS technologies will reveal more genetic diversity for many commercial breeds with short turnaround time. For example, NGS can help to sequence mutant lines in a much more efficient manner. By identifying genes/proteins with desirable traits (disease resistance and/or high milk/egg/meat production), researchers could better control selection, and this will in turn improve both productivity and animal welfare. Sequencing individual agricultural animals will increase opportunities for resisting animal pathogens that can challenge meat/egg/dairy production. Since domestic animals are the leading source of animal protein for human beings, the sequencing research will provide valuable information for efficient production of a leaner, healthier and more economical source of animal protein for human consumption.

The breeding of farm animals is entering the post-genome era [32]. Despite some deficiencies of NGS, e.g. poor coverage of GC rich areas and the challenges in the assembly when a good reference genome is not available, NGS technologies (RNA-Seq, Chip-Seq, and Genome-resequencing) are still able to help animal scientists study individual genomes at a pace far quicker than previously could be achieved. We believe that sequencing individual animals treated with different conditions shows great promise. Sequencing micro-organisms and parasites in agricultural animals' organs can also help veterinarians develop new vaccines and therapeutics [32]. NGS will also facilitate the study of gene expression and regulatory mechanisms of milk production and egg/meat flavor in animals. By utilizing NGS approaches/tools, researchers can identify and further analyze individual genes controlling/affecting economic traits in agricultural animals, which will eventually benefit the consumers.

## Abbreviations

This is a list of abbreviations used in the text: NGS: Next Generation Sequencing; NCBI: National Center for Biotechnology Information; SNP: Single Nucleotide Polymorphism; BBSRC: Biotechnology and Biological Sciences Research Council; EST: Expressed Sequence Tag; cDNA: Complementary Deoxyribonucleic Acid; QTL: Quantitative Trait Loci; bp: base pair; Gb: Gigabyte; BAC: Bacteria Artificial Chromosomes; UMD: University of Maryland; WGS: Whole Genome Shotgun.

## Competing interests

The authors declare that they have no competing interests.

## Authors' contributions

YB carried out the review studies and drafted the manuscript. MS and JC participated in drafting the manuscript. All authors read and approved the final manuscript.
